# Translocator protein PET imaging in temporal lobe epilepsy: A reliable test-retest study using asymmetry index

**DOI:** 10.3389/fnimg.2023.1142463

**Published:** 2023-04-28

**Authors:** Mohammad Mahmud, Charles Wade, Sarah Jawad, Zaeem Hadi, Christian Otoul, Rafal M. Kaminski, Pierandrea Muglia, Irena Kadiu, Eugenii Rabiner, Paul Maguire, David R. Owen, Michael R. Johnson

**Affiliations:** ^1^Department of Brain Sciences, Imperial College London, London, United Kingdom; ^2^Clinical Imaging Translational, UCB Pharma SA, Brussels, Belgium; ^3^Department of Medicinal Chemistry, Faculty of Pharmacy, Jagiellonian University Medical College, Krakow, Poland; ^4^GRIN Therapeutics, Brussels, Belgium; ^5^Translational Applications, Invicro LLC, London, United Kingdom

**Keywords:** epilepsy, TSPO, PET, neuroinflammation, hippocampus, seizure, test–retest

## Abstract

**Objective:**

Translocator protein (TSPO) targeting positron emission tomography (PET) imaging radioligands have potential utility in epilepsy to assess the efficacy of novel therapeutics for targeting neuroinflammation. However, previous studies in healthy volunteers have indicated limited test-retest reliability of TSPO ligands. Here, we examine test-retest measures using TSPO PET imaging in subjects with epilepsy and healthy controls, to explore whether this biomarker can be used as an endpoint in clinical trials for epilepsy.

**Methods:**

Five subjects with epilepsy and confirmed mesial temporal lobe sclerosis (mean age 36 years, 3 men) were scanned twice—on average 8 weeks apart—using a second generation TSPO targeting radioligand, [^11^C]PBR28. We evaluated the test-retest reliability of the volume of distribution and derived hemispheric asymmetry index of [^11^C]PBR28 binding in these subjects and compared the results with 8 (mean age 45, 6 men) previously studied healthy volunteers.

**Results:**

The mean (± SD) of the volume of distribution (*V*_T_), of all subjects, in patients living with epilepsy for both test and retest scans on all regions of interest (ROI) is 4.49 ± 1.54 vs. 5.89 ± 1.23 in healthy volunteers. The bias between test and retest in an asymmetry index as a percentage was small (−1.5%), and reliability is demonstrated here with Bland-Altman Plots (test mean 1.062, retest mean 2.56). In subjects with epilepsy, *V*_T_ of [^11^C]PBR28 is higher in the (ipsilateral) hippocampal region where sclerosis is present than in the contralateral region.

**Conclusion:**

When using TSPO PET in patients with epilepsy with hippocampal sclerosis (HS), an inter-hemispheric asymmetry index in the hippocampus is a measure with good test-retest reliability. We provide estimates of test-retest variability that may be useful for estimating power where group change in *V*_T_ represents the clinical outcome.

## 1. Introduction

Epilepsy affects approximately 1% of the population globally (around 50 million people worldwide) and is a serious neurological disorder characterized by a predisposition to spontaneous epileptic seizures. Epilepsy also has significant consequences psychologically, cognitively, and neurobiologically (Fisher et al., 2014). Mesial temporal lobe epilepsy is the most prevalent form of chronic epilepsy, and hippocampal sclerosis (HS) is the most common focal pathology and is the primary histopathology found in over one-third of adult patients undergoing epilepsy surgery (Blumcke et al., 2017). The current use of anti-epileptic drugs in patients with epilepsy is aimed at reducing or stopping seizures, but as yet, treatments do not affect the underlying etiology of the disease and are ineffective in one-third of patients (Perucca et al., 2007).

The innate immune system has been implicated in epileptogenesis and ictogenesis (Vezzani et al., 2019). Animal studies and human pathology studies of subjects with temporal lobe epilepsy support the role of several immune and inflammatory pathways, including IL-1R/TLR signaling, COX-2 signaling, tumor necrosis factor alpha, complement system, and chemokines (Vezzani et al., 2011, 2019; Aronica et al., 2012; Librizzi et al., 2012). These existing empirical data suggest various molecular roles that lead to seizure onset and recurrence. These mechanisms include alterations to activated microglia and astrocytes, modifications in glutamine and glutamate cycles and receptor expression, and neuromodulatory release of the inflammatory pathways mentioned. Anti-inflammatory drugs targeting these pathways and gene-regulatory networks have since been seen as a potential therapeutic approach in patients with epilepsy and roles for biomarkers of these pathways have also been considered (Johnson et al., 2015; Srivastava et al., 2018; Vezzani et al., 2019). A method of reliably measuring the anti-inflammatory effect of novel treatments would be very useful in clinical development programs (Scott et al., 2017).

The most commonly used method to quantify inflammation in the living human brain is positron emission tomography (PET), targeting the translocator protein 18 kDa (TSPO), which has been studied in both animal models and in subjects with epilepsy (Hirvonen et al., 2012; Xie et al., 2012; Gershen et al., 2015; Yankam Njiwa et al., 2017; Nguyen et al., 2018). TSPO is expressed at low levels in the central nervous system, but its expression is increased as microglia proliferate and peripheral myeloid cells are recruited (Gerhard et al., 2005; Liu et al., 2014). PET with TSPO showed an association with microglial activation, astrogliosis, and cell death. So far, imaging of neuroinflammation in the context of epilepsy supports the view that activated microglia, reactive astrocytes, and inflammatory intermediates may contribute to hyperexcitability in seizure foci. Invasive studies using TSPO tracers [^11^C]-(R)-PK11195 and [^11^C]PBR28 indicate a specific and consistent increase in the numerical density of activated microglia in dysplastic regions of patients with epilepsy (Butler et al., 2013; Gershen et al., 2015). Since the development of the first generation TSPO PET radioligand, [^11^C]PK11195, second generation radioligands have been developed that improve the signal-to-noise ratio. Quantitative interpretations of the signal are however confounded by large interindividual variability in binding affinity, and previous work has revealed that TSPO ligands show substantial differences in affinity between subjects classified as high-affinity binders (HABs) and low-affinity binders (LABs) (Owen et al., 2010, 2011, 2012).

In order to be able to understand how much of the overall variability can be attributed to biological factors, there is a need to understand the intra-subject test-retest reliability of putative markers. Recently, Dickstein et al. attributed the lack of significant TSPO absolute binding measurement differences between subjects with neocortical seizure foci and healthy controls to heterogeneity, perhaps indicating that larger sample sizes are required to detect smaller differences (Dickstein et al., 2019).

The [^11^C]PBR28 PET signal has been studied previously in 16 subjects with temporal lobe epilepsy and showed a higher ipsilateral brain uptake of the radioactive signal corresponding with the side of the seizure focus (Hirvonen et al., 2012). Specifically, the brain uptake of the radioactive signal in specific regions of interest (ROI), including hippocampal, parahippocampal, gyrus, amygdala, fusiform gyrus, and choroid plexus, was higher ipsilaterally (4–16%). This hemispheric asymmetry was more pronounced in the nine subjects who had MRI-confirmed hippocampal sclerosis (mean ± SD; 13 ± 6% compared to 5 ± 4%). Interhemispheric comparison of TSPO binding is a useful approach to reduce the confounding factors of PET studies, including controlling for individual variations of demographics and medications, and appears to have been sensitive in that study. However, intra-subject variability in the TSPO signal was not studied in the subjects with temporal lobe epilepsy as each subject only had one scan (Hirvonen et al., 2012). In another study by Gershen et al., PET imaging was studied in subjects with temporal lobe epilepsy using two different radioligands; [^11^C]PBR28 (23 subjects) and [^11^C]DPA-713 (8 subjects) alongside aged-matched healthy controls for both groups (Gershen et al., 2015). In all subjects with temporal lobe epilepsy, a hemispheric asymmetry was seen, with higher uptake in the ipsilateral hemisphere to the seizure foci, and this asymmetry was significant (*p* < 0.05) in the 10 subjects who had confirmed hippocampal sclerosis. Although, as mentioned above, Dickstein et al. were unable to show significant TSPO absolute binding measurement, 9 out of 11 patients had asymmetry indexes exceeding mean 95% confidence intervals for 31 controls in the anatomic region of interest, which is consistent with the seizure focus (Dickstein et al., 2019).

These studies suggest that TSPO PET may be a useful tool to assess candidate molecules for disease modulation in epilepsy based on novel therapeutics targeting inflammatory processes. However, the intra-subject (test-retest) variability in the TSPO signal is unknown in epilepsy, and studies in healthy volunteers suggest that the test-retest variability is limited (Park et al., 2015; Collste et al., 2016; Nair et al., 2017). Test-retest variability is critical to accurately calculate sample size for an interventional study. Therefore, using [^11^C]PBR28, we scanned subjects with epilepsy with confirmed hippocampal sclerosis twice over 8 weeks to determine the test-retest reliability.

## 2. Methods

### 2.1. Study methodology and participants

This study included eight (mean age 45, 6 men) previously studied healthy volunteers and compared to five subjects (3 men) aged between 28 and 50 years with clinically definite mesial temporal lobe sclerosis and electroencephalographically confirmed epilepsy after evaluation by an epileptologist (principal investigator) and a neuroradiologist. Patients were recruited from the Imperial College Healthcare NHS Trust. All subjects gave written consent, and their eligibility was confirmed through a full medical history, physical and neurological examinations, routine blood tests, and electrocardiogram. Exclusion criteria included other focal lesions and serious medical conditions, pregnancy, and a history of substance abuse. All subjects received a high-resolution T1 magnetic resonance imaging (MRI) scan in a Siemens Tim Trio 3T scanner (Siemens Healthcare, Erlangen, Germany), which was used to re-confirm evidence for hippocampal sclerosis. All subjects had two [^11^C]PBR28 PET scans in a Siemens Biograph 6 PET-CT with Truepoint gantry 8 weeks apart, and their seizure activity was reported over this period. All structural magnetic resonance images were inspected by a neuroradiologist for unexpected findings of clinical significance or features that might confound PET co-registration or quantitative analysis. These results were compared to eight healthy control subjects that were age and sex matched (of which only five healthy volunteers had a *V*_T_ 2TC analysis). The second generation radioligands, such as [^11^C]PBR28, are complicated by the common polymorphism (rs6971) in the TSPO gene which produces three classes of binding affinity in the general population: high-affinity binders (HAB), mixed-affinity binders (MAB), and low-affinity binders (LAB) (Owen et al., 2010, 2012). All subjects were genotyped for rs6971 polymorphism using a Taqman SNP Genotyping Assay. Subjects with the LAB genotype were excluded from the study because of a negligible specific signal.

### 2.2. Preparation of [^11^C]PBR28 radiopharmaceutical preparation

Cyclotron-produced [^11^C]CO_2_ was converted to [^11^C]methyl iodide using a GE Microlab system (GE, Uppsala, Sweden). The [^11^C]methyl iodide was subsequently passed through a solution containing 1 mg of desmethyl-PBR28 in 350 μl DMF, and 0.5 M tetrabutyl ammonium hydroxide in methanol (4 μl) as the base. The reaction mixture was heated to 120°C for 5 min, allowed to cool, and diluted with high-performance liquid chromatography (HPLC) solvent [ammonium formate 10 mmol/L: acetonitrile 57/43 (v/v), 8 ml/min]. The fraction containing [^11^C]PBR28 was collected into a vessel containing 20 ml water for injection before trapping onto a solid-phase cartridge (C18 Classic SepPak, Waters, Milford, MA, USA). The cartridge was washed with water for injection, and [^11^C]PBR28 was eluted using ethanol (1 ml) followed by saline for injection (9 ml) through a 0.2 μm sterile filter (Pall Acrodisc, Sterile, 33 mm, 0.22 μm, Pall, Port Washington, NY, USA) into a sterile vial (Adelphi, Manchester, UK).

### 2.3. Quality control

For the following section, (A) indicates tests performed as part of the release process, and (P) indicates tests performed as part of the post release processes. The final product was tested using validated procedures in accordance with good manufacturing practices for the following: visual appearance clear, colorless, and practically free from particles (A), pH between 4.5 and 8.5 (A), total endotoxins <175EU/dose (A), PBR28 mass ≤ 10 μg per dose (3.43 ± 1.69 μg), desmethyl-PBR28 <1 μg per dose, total unknown chemical impurities ≤ 12 μg per dose (A), radiochemical purity >95% (A), radiochemical identity co-elution with cold PBR28 (A), isotopic half-life within 10% of expected value (A), filter integrity pass (A), isotopic purity (no additional gamma peaks in a fully decayed sample) (P), residual solvents within limits set by International Conference on Harmonization Q3C R4 (P), and sterility confirmed (P). All batches used for this study successfully passed the specifications listed above.

### 2.4. Analysis of [^11^C]PBR28 plasma metabolism

The [^11^C]PBR28 parent fraction over the course of the PET scan was determined using the “Hilton method” (Owen et al., 2014). Briefly, urea was added to the plasma samples to give an 8 mol/L concentration to break up plasma-protein binding. The sample was diluted 1:1 with 0.1 mol/L unbuffered Tris and filtered through a 0.45 μm polyvinylidene fluoride syringe filter (Waters Acrodisc LC 13 mm Minispike and 25 mm, Waters, USA) before injecting onto the dual HPLC system. System 1 operated as a trapping system where parent and non-polar metabolites were collected on a Biotrap 500 MS (ChromTech, Cedex, France) sample-enriching column using 5% acetonitrile in 0.1 mol/L unbuffered Tris as solvent at a flow rate of 2 ml/min. Following trapping, the Biotrap column was switched in line with HPLC system 2. Parent and non-polar metabolites were eluted using a gradient method (0–4 min 5% acetonitrile: 95% unbuffered Tris, 4–10 min 5% acetonitrile → 95% acetonitrile) and separated using an Agilent Zorbax Eclipse XDB-C18 column (5 μm, 150 × 4.6 mm, Agilent). A total of 30 fractions were collected, and activity was measured in a Perkin Elmer 1470 10-well-automated gamma counter (Perkin Elmer, Turku, Finland).

### 2.5. Positron emission tomography data acquisition

The [^11^C]PBR28 was injected as an intravenous bolus over ~20 s at the start of a 90-min 3D-mode dynamic PET acquisition. Injected activities ranged from 292 to 357 MBq (Mean ± SD; 333 ± 16 MBq, *n* = 26). Positron emission tomography data were reconstructed using filtered back projection with corrections for attenuation and scatter (based on a low-dose CT acquisition). Dynamic data were binned into 26 frames (durations: 8 × 15 s, 3 × 1 min, 5 × 2 min, 5 × 5 min, and 5 × 10 min). Arterial blood samples were collected from the radial artery to enable the generation of an arterial plasma input function. A continuous sampling system (ABSS Allogg, Mariefred, Sweden) was used to measure whole blood activity each second for the first 15 min of each scan. Discrete blood samples were manually withdrawn at 5, 10, 15, 20, 25, 30, 40, 50, 60, 70, 80, and 90 min after the start of the scan to facilitate the measurement of whole blood and plasma activity. Samples taken at 5, 10, 20, 30, 50, 70, and 90 min time points were also analyzed using HPLC to determine the fraction of parent radioactivity in arterial plasma.

The procedure used to generate the metabolite corrected plasma input function has been described earlier and in previous studies (Owen et al., 2014). In brief, the total plasma curve was generated by multiplying the whole blood curve by the plasma-over-blood ratio, and the parent fraction data were fitted to a sigmoid model: where *t* is time, and *a, b*, and *c* are fitted parameters.


f=((1−t+10a)b+c)/(1+c).

The resulting fitted parent fraction profile was multiplied by the total plasma curve and then smoothed post peak using a tri-exponential fit to derive the required parent plasma input function.

### 2.6. Image analysis

Motion in the dynamic PET data was corrected *via* frame-to-frame image registration of the non-attenuation corrected PET image to the individual's structural T1 magnetic resonance image using SPM5 (The Wellcome Centre for Human Neuroimaging, http://www.fil.ion.ucl.ac.uk/spm) with a mutual information cost function (see image example in Figure S1). The CIC neuroanatomical atlas was non-linearly deformed into the individual's space, *via* T1 magnetic resonance imaging data mapping, to obtain a personalized anatomical panellation of ROIs (Owen et al., 2014). Attention focused on regions of different levels of binding are as follows: frontal, occipital, parietal, temporal, and cingulate cortex, hippocampus, thalamus, striatum, putamen, cerebellum, brainstem, midbrain, and pons. Cortical gray matter, whole gray and white matter, and the whole brain were also included in the region of interest (ROI) analysis. Each ROI was then applied to the dynamic PET data to derive regional time-activity curves.

### 2.7. Kinetic analysis and data analysis

A two tissue-compartment model, utilizing the metabolite corrected plasma input function, has been shown previously to be a suitable model and was applied to the dynamic PET data using a fixed blood volume correction of 5% (Owen et al., 2014). A time delay correction was performed for each scan to account for any temporal delay between blood sample measurement and the tomographic measurements of the tissue data. For each ROI examined, the *V*_T_ was estimated from the rate constants as described previously (Gunn et al., 2000; Innis et al., 2007). Model fitting and parameter estimation were performed using software implemented in Matlab R2008b (The MathWorks, Natick, MA, USA). *V*_T_ was not corrected for measured free fraction; the free fraction is on average 2% of the total but is unreliable to measure. A within-subject normalized *V*_T_ was calculated by dividing regional *V*_T_ by average *V*_T_ over six regions (the frontal lobe, parietal lobe, occipital lobe, cerebellum, midbrain, and thalamus).

The asymmetry index is defined as the ratio of outcome parameter comparing the ipsilateral (epileptic focus) to the contralateral side. To compare calculated asymmetry indices between patients with epilepsy, we used the following formula:


$$\begin{array}{l} \left[\text{AIs};200{\%}^{*}(\text{ipsilateral}-\text{contralateral})/(\text{ipsilateral}\right)\\ \;+\text{contralateral})]. \end{array}$$

*V*_T_ data and asymmetry index data were analyzed using Microsoft EXCEL and using SAS^®^ version 9.4 (SAS Institute, Cary, NC, USA). Descriptive statistics and graphical display methods were applied to summarize these data. Paired *t*-test and linear mixed model were used to evaluate differences and estimate least squares means for the different effects. On the asymmetry index, an ANOVA linear model was applied to evaluate the effect of ROI and scans as a fixed factor. The interaction between the two factors was also assessed. A two-sided *p*-value of 0.05 was used as the threshold of statistical significance for testing comparison, and 95% confidence intervals were applied. Bland-Altman plots were used to graphically display the level of agreement between the test and retest.

## 3. Results

All five subjects with epilepsy completed both scans 8 weeks apart. Their seizure activity during this time is reported in Table 1. Their mean ± SD age was 36.4 (±10.4) years. The mean age of eight healthy controls was 45 (±19.2) years. There were no significant differences in the demographic characteristics between our subjects and the healthy controls.

**Table 1:** Demographics of subjects with epilepsy, seizure frequency within last 8 weeks, and medications from 8 weeks prior to imaging and remained unchanged between imaging.

**Subject number**	**Gender**	**Age (years)**	**TSPO affinity**	**Side of hippocampal sclerosis**	**Seizure frequency (8 weeks)**	**Medications**	**Injected radioligand (MBq/μg) TEST**	**Injected radioligand (MBq/μg) RETEST**
**1**	F	31	High (HAB)	Left	4x Focal loss of awareness 1x GTCS	Lacosamide 125 mg BD	308/4.34	112/8.58
**2**	M	28	High (HAB)	Right	2x GTCS	Sodium valproate 900 mg BD	314/4.53	334/5.41
**3**	M	45	High (HAB)	Right	9x Focal loss of awareness	Levetiracetam 500 mg BD	299/2.05	138/7.76
**4**	F	50	High (HAB)	Left	1x Focal loss of awareness 2x Nocturnal generalized	Lacosamide 100 mg BD Topiramate 275 mg BD Carbamazepine 400 mg BD	204/8.70	301/2.85
**5**	M	28	Moderate (MAB)	Left	1x Focal loss of awareness	Levetiracetam 1,500 mg BD Carbamazepine 500 mg BD	332/1.09	365/2.58

Each subject's scan injected activities are shown as a Megabecquerel (MBq) and mass (μg). GTCS, generalized tonic clonic seizure; BD, twice daily; OD, once daily; F, female; M, male; HAB, high affinity binders.

TSPO genotyping in the five subjects with epilepsy demonstrated 4 HABs and 1 MAB. In healthy controls, there were 6 HABs and 2 MABs. Following intravenous injection, [^11^C]PBR28 appeared rapidly in the brain and was homogenously distributed across all brain regions. Injection parameters were similar between the test and retest scans (*p* = 0.51). The mean ± SD injected dose was 291 ± 50 MBq for the test and 250 ± 117 MBq for the retest (*p* = 0.24). The mean ± SD injected mass was 4.14 ± 2.94 μg for the test and 5.44 ± 2.74 μg for the retest (*p* = 0.24).

**Figure 1:**
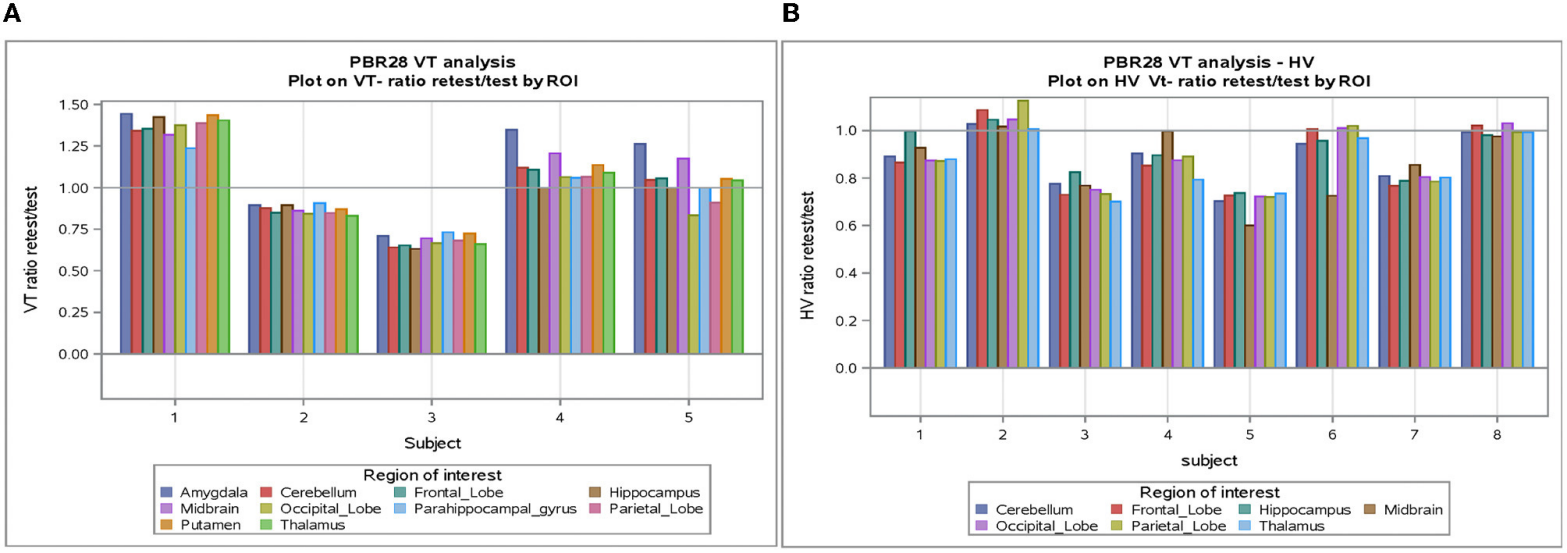
**(A)** V_T_-Ratio test/retest in five subjects with epilepsy by ROI. **(B)** V_T_-Ratio test/retest in eight of the healthy volunteers by ROI. ROI, Region of interest; V_T_, volume of distribution; HV, healthy volunteer.

**Figure 2:**
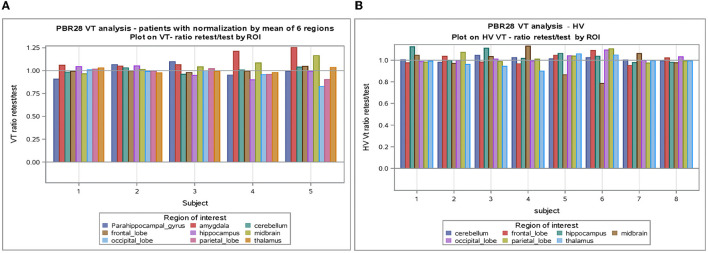
**(A)** V_T_ normalized by mean of six regions in subjects with epilepsy. **(B)** V_T_ normalized by mean of six regions in healthy volunteers. ROI, Region of interest; V_T_, volume of distribution; HV, healthy volunteer.

The individual volume of distribution (*V*_T_) in the regions of interest varied between 1.8 and 7.9 (Table 2) in subjects with epilepsy (between 3.04 and 10.07 in healthy volunteers). The overall mean (± SD) of all subjects with epilepsy for both test and retest scans on all regions of interest is 4.49 ± 1.54 vs. 5.89 ±1.34 in healthy volunteers. The between subject results for each scan in subjects with epilepsy (test and retest) were, respectively, 4.55 ± 1.64 and 4.42 ± 1.44 (Table 3). The *V*_T_ results for each scan in healthy volunteers (test and retest) were, respectively, 6.33 ± 1.22 and 5.45 ± 1.33 (Table 3). The normalized *V*_T_ and test/retest ratio of *V*_T_ in subjects with epilepsy and healthy volunteers are shown in Figures 1, 2, respectively. The estimated difference in *V*_T_ (within subject) from the ANOVA model for subjects with epilepsy was estimated to be 0.13 ± 0.69 and 0.88 ± 0.30 for healthy subjects.

**Table 2:** The test-retest variability of subjects with epilepsy represented as a percentage within each area of interest.

**Subject number**	**Frontal lobe (%)**	**Parietal lobe (%)**	**Occipital lobe (%)**	**Cerebellum (%)**	**Putamen (%)**	**Midbrain (%)**	**Thalamus (%)**	**Hippocampus (%)**	**Amygdala (%)**	**Parahippocampal gyrus (%)**
**1**	30	32	29	31	36	28	34	34	35	22
**2**	15	18	16	14	15	15	18	12	12	10
**3**	41	38	39	44	32	36	40	44	35	31
**4**	9	7	6	12	14	18	9	0	29	5
**5**	3	8	17	5	3	14	5	2	21	3
***Mean***	*20*	*21*	*22*	*21*	*20*	*22*	*21*	*18*	*26*	*14*
***SD***	*16*	*14*	*13*	*16*	*14*	*10*	*15*	*20*	*10*	*12*

Total volume of distribution (*V*_T_) equivalent to an absolute measure of total ligand binding (displaceable + non-displaceable + free).

**Table 3:** V_T_, V_T_ normalized, and asymmetry data for subjects with epilepsy and healthy volunteers.

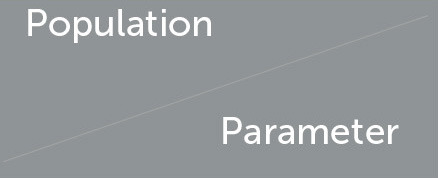	**Assessment**	**Descriptive statistics Mean**	**SD**	**Pearson coefficient of correlation (test/retest)**	**Estimated mean difference and SD within subject (test vs. retest) from ANOVA**
**Epilepsy subjects (*****N*** = **5)**					
***V_T_*** **(*****n*****=** **50 per scan)**	Test	4.55	1.64	0.744 (*p* < 0.001)	0.13 (0.69)
	Retest	4.42	1.44		
	Overall (test + retest)	4.49	1.54		
***V_T_*** **normalized (*****n*** **=** **45 per scan)**	Test	1.013	0.105	0.788 (*p* < 0.001)	–0.012 (0.065)
	Retest	1.025	0.120		
	Overall	1.019	0.112		
**Asymmetry index (%) (*****n*** **=** **35 per scan) Using 7 ROIs**	Test	1.062%	16.50%	0.120 (*p* = 0.491)	–1.5% (12.75 %)
	Retest	2.562%	9.33%		
	Overall (test + retest)	1.812%	13.33%		
**Asymmetry index (ratio) (*****n*** **=** **35 per scan)**	Test	1.021	0.141	0.197 (*p* = 0.256)	−0.01 (0.11)
	Retest	1.030	0.095		
	Overall (test + retest)	1.026	0.118		
**Healthy subjects (*****N*** = **5 for** *V*_T_**; 8 for** *V*_T_ **normalized)**					
***V_T_*** **(*****n*** **=** **40 per scan)**	Test	6.33	1.22	0.697 (*p* < 0.001)	0.88 (0.30)
	Retest	5.45	1.33		
	Overall (test + retest)	5.89	1.34		
***V_T_*** **normalized (*****n*** **=** **56 per scan) Using 7 ROIs**	Test	0.999	0.141	0.871 (*p* < 0.001)	–0.005 (0.063)
	Retest	1.005	0.118		
	Overall (test + retest)	1.002	0.130		

ROI, region of interest; V_T_, volume of distribution. The asymmetry index (%) is calculated as [200 ^*^ (C – I)/(C + I)].

The SD between subjects with epilepsy was estimated as 1.49 (1.39 in healthy) with a coefficient of variation of 33% (24% in HV), while the SD within subjects with epilepsy (test-retest) was estimated as 0.69 (0.30 in HV) with a coefficient of variation of 15% (5% in HV).

The correlation between test and retest *V*_T_ values was consistent [Pearson coefficient of correlation was 0.744 (*P* < 0.001)] in subjects with epilepsy and in healthy volunteers [Pearson coefficient of correlation was 0.697 (*P* < 0.001)] (see Table 3 and Figure S3). This agreement (reliability) in both subjects with epilepsy and healthy volunteers between test-retest is highlighted in the Bland-Altman plots with a small bias and two outliers beyond the 95% confidence intervals.

An analysis of *V*_T_ normalized in patients with epilepsy and healthy volunteers showed again good test-retest correlation (*r*) (*r* = 0.788 and *r* = 0.871, respectively, *p* < 0.0001) for both (Table 3). When normalization is applied on *V*_T_, the coefficient of variation dropped to 5–6% between subjects and 2–4% within subjects. Encouraging the use of normalization, Figure 2 shows that the *V*_T_-ratio test/retest is consistent within subject, across ROIs, suggesting that one part of the overall variance is an effect across all ROIs.

There was a statistically significant difference between the different ROIs with the highest means for the midbrain and thalamus and lowest means for the frontal and parietal lobe, while there was no significant interaction between the scan and ROI (*p* = 0.998).

In all five subjects with epilepsy, in the hippocampal region where the sclerosis is present (ipsilateral), the ipsilateral *V*_T_ is higher than contralateral *V*_T_, and this had good test-retest reliability (mean 18% and SD 20%, as shown in Table 2).

**Figure 3:**
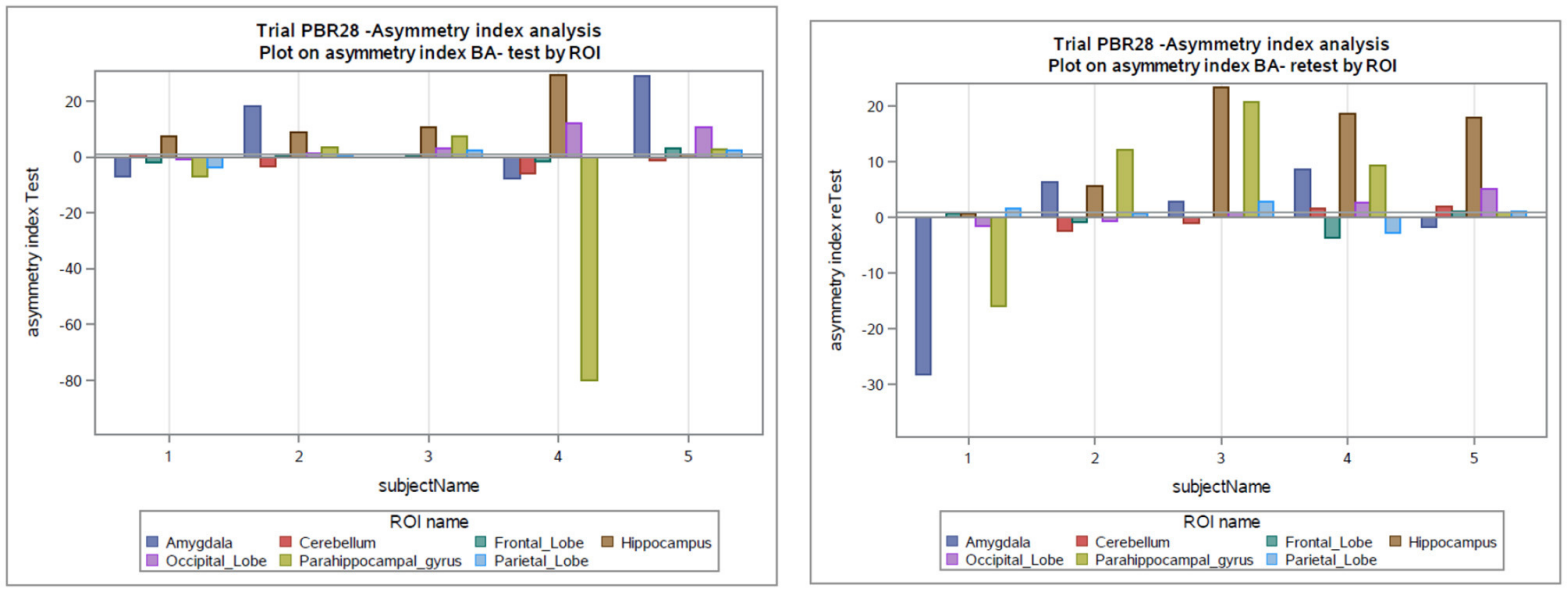
Asymmetry index as a percentage in the test and retest scans in subjects with epilepsy. BA, asymmetry index as Bland-Altman (BA) formula; ROI, Region of interest.

The global mean hippocampal asymmetry and its imprecision are tabulated for the regions of interest (Table 4). The asymmetry index analysis in subjects with epilepsy is shown in Table 3 and Figure 3. The bias between test and retest in an asymmetry index as a percentage was −1.5% (SD 13%, min −80%, and max 29.55%) compared to the global mean hippocampal asymmetry, and the reliability is seen on the Bland-Altman plot (Figure S2). Considering the precision of the test and retest over all ROIs, the SD within epilepsy subjects (test-retest) was estimated to be 12.75% for the asymmetry index in terms of % and 0.11 when the index is calculated as a ratio (Table 3). The Pearson correlation coefficient for the test-retest reliability was 0.197 (*p* = 0.256). An ANOVA on asymmetry index—comparison of ROI (2TC) revealed statistically significant differences of the least square means between the hippocampus and all other ROIs. However, ROIs were not significantly different from each other. Generalized seizure counts significantly correlated with the asymmetry index in the frontal (−0.662, *p* = 0.037) and parietal (−0.740, *p* = 0.015) lobes.

**Table 4:** The global mean asymmetry of the hippocampus at each ROI as a mean percentage in test- retest and the imprecision (mean difference).

**Region of interest**	**Mean test (%)**	**Mean retest (%)**	**Mean difference (%)**
Amygdala	6.58	−2.42	9
Cerebellum	−1.94	0.05	−1.99
Frontal lobe	0.22	−0.49	0.71
Hippocampus	11.5	13.28	−1.78
Occipital lobe	5.32	1.31	4.01
Parahippocampal gyrus	−14.65	5.5	−20.15
Parietal lobe	0.41	0.71	−0.3

However, total, generalized, or focal seizure counts did not significantly correlate with the asymmetry index in other ROIs. This last analysis is based on the small sample size.

## Discussion

Our results demonstrate significant inter-hemispheric differences in patients with epilepsy and hippocampal sclerosis and indicate that the test-retest of the hippocampal asymmetry index is a reliable measure for future research, more so than the test-retest of *V*_T_ alone, which likely will necessitate a larger sample size. This reliability is critical for any interventional study aimed at showing on-target drug effects on neuroinflammation related to the seizure focus.

Previous studies looking at hemispheric asymmetry in subjects with epilepsy using [^11^C]PBR28 PET imaging have shown higher ipsilateral brain uptake of the radioactive signal corresponding with the side of the seizure focus, but it was more pronounced in nine out of sixteen subjects with hippocampal sclerosis (Hirvonen et al., 2012). With the same group of subjects, it was demonstrated with PET imaging that subjects with temporal lobe epilepsy had higher uptake in the ipsilateral hemisphere to the seizure foci, and this asymmetry was once again more significant in the subjects with confirmed hippocampal sclerosis (Gershen et al., 2015). The test-retest of the hippocampal asymmetry index is shown here to be good enough to make this imaging technique usable, and certainly appears more reliable than *V*_T_ variability alone.

Our subjects also demonstrated higher ipsilateral brain uptake of the radioactive signal corresponding with the side of the seizure focus and hippocampal sclerosis (despite decreased brain volume secondary to atrophy). Previous attempts to provide a test-retest measure in PET brain imaging have been difficult due to the large inter-individual variability (Owen et al., 2012; Collste et al., 2016). This variability is only partly reduced by ensuring that diurnal variation is accounted for. Therefore, the use of an asymmetry index as a laterality ratio in a region of interest with a test and retest scan produces higher reliability.

This study adds weight to the argument that the hippocampal asymmetry index is clinically meaningful (i.e., it correlates with seizure frequency). The seizure frequency correlated with *V*_T_ in this study of only five patients. A previous study by Butler et al. suggested an increased TSPO PET tracer uptake and spatial extent in one patient post seizure (Butler et al., 2016). We also explored the relation of uptake of radioactive signal to patient's age, inter-scan seizure frequency, and *V*_T_ distribution or asymmetry index but did not find a strong correlation, similar to a previous study (Dickstein et al., 2019).

Caution is advised when interpreting PET brain imaging studies such as ours. The 18 kDa Translocator Protein (TSPO) is the most commonly used tissue-specific marker of inflammation in positron emission tomography (PET) clinical studies (Vivash and O'Brien, 2016). When interpreting studies using TSPO signaling in the brain uptake, it is important to note that the signal is non-specific due to the mixture of cells (Owen et al., 2012). Owen et al. have also demonstrated in *in vitro* studies that increases in the TSPO PET specific signal in humans may reflect either local myeloid cell proliferation or monocyte recruitment rather than microglial activation. Moreover, these considerations do not account for the potential role of astrocytes in TSPO expression. A more recent study exploring TSPO expression in basal conditions in hippocampal cells of mouse models corroborates the evidence of the multi-cellular expression profile of TSPO, in which a causal relationship is indicated for neuronal activation as a non-inflammatory mechanism and altered TSPO levels (Notter et al., 2020). Our study has several limitations including a small and conservative sample size with subjects of varied disease duration, age, seizure frequency, antiepileptic drugs, and affinity of TSPO binding. In conclusion, however, this study does show good test-retest reliability using a hemispheric asymmetry index in subjects with epilepsy with hippocampal sclerosis, which may make it a suitable intermediate imaging endophenotype.

## Data availability statement

The original contributions presented in the study are included in the article/Supplementary material, further inquiries can be directed to the corresponding author.

## Ethics statement

The studies involving human participants were reviewed and approved by West London Research Ethics Committee and ARSAC (Administration of Radioactive Substances Advisory Committee). The patients/participants provided their written informed consent to participate in this study.

## Author contributions

MM: data curation, formal analysis, investigation, methodology, project administration, roles/writing—original draft, and review and editing. CW, SJ, and ZH: formal analysis, writing—review, and editing. CO: investigation, methodology, validation, visualization, writing—review, and editing. RMK, PMu, and IK: conception, methodology, project administration, writing—review, and editing. ER, PMa, DO, and MJ: conception, supervision, funding, writing—review, and editing. All authors contributed to the article and approved the submitted version.
