# Supplementary material for: Translocator protein PET imaging in temporal lobe epilepsy: A reliable test-retest study using asymmetry index
Source: Front Neuroimaging. 2023 Apr 28;2:1142463. doi: 10.3389/fnimg.2023.1142463 (PMC10406252; doi:10.3389/fnimg.2023.1142463)
Supplement: Supplementary file 1 [file Data_Sheet_1.docx]

1. **Supplementary**


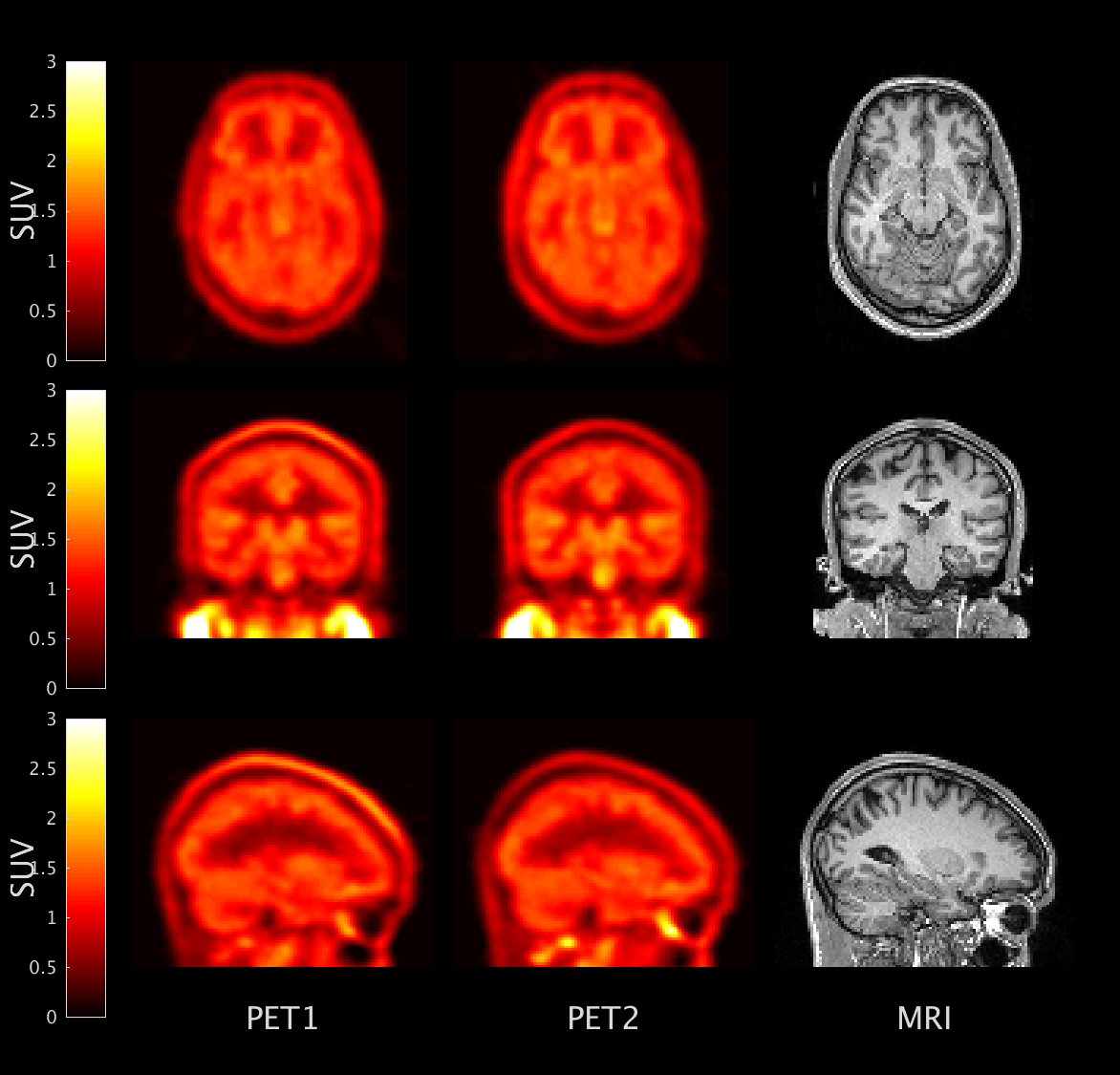


Figure S1 – Subject 2 integral images of PET and MRI brain. A subject with mesial temporal lobe sclerosis. Standardised uptake value (SUV) comparing the PET imaging from the test and retest scans in three planes.


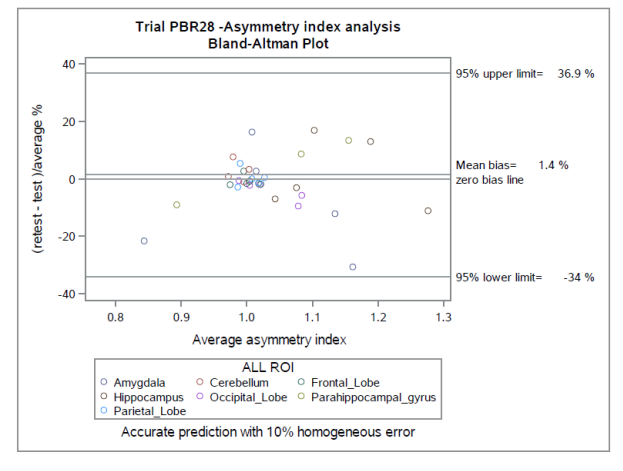


Figure S2 - Bland-Altman plot of epilepsy patients test/retest agreement in asymmetry index as a ratio in subjects with epilepsy; BA= Bland-Altman; ROI = Region of interest.


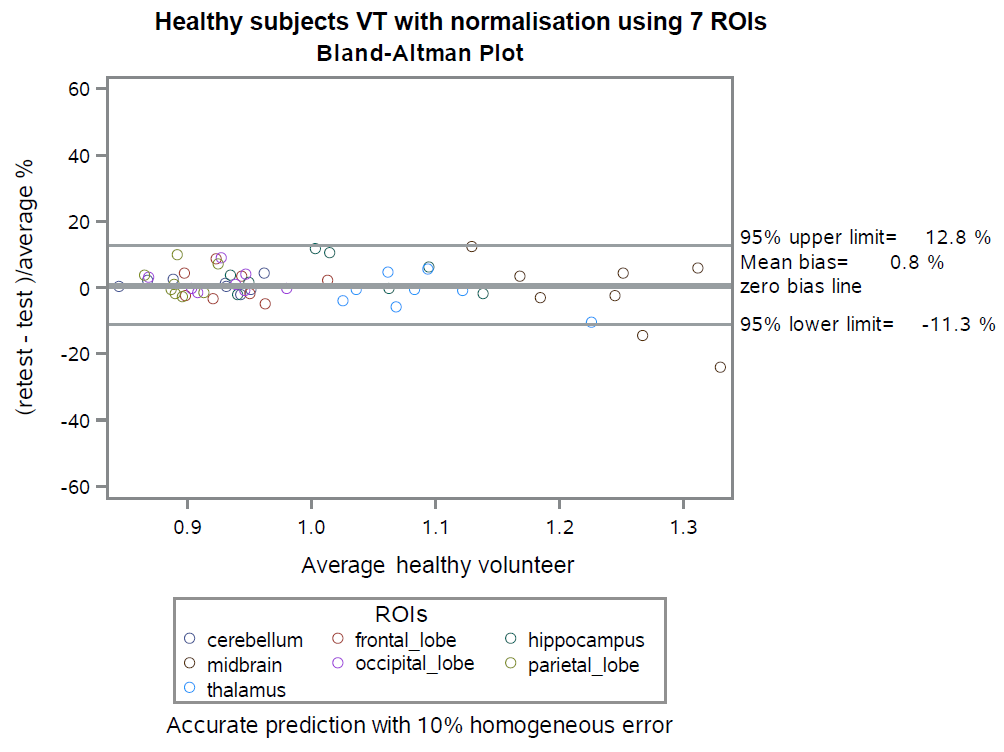


Figure S3 - Bland-Altman plot of healthy subjects VT data with normalisation using 7 ROIs (n=56 per scan) ROI = Region of interest; VT = volume of distribution.

Figure S4 - Bland-Altman plot of healthy subjects VT data without normalisation using 7 ROIs (n=40 per scan). ROI = Region of interest; VT = volume of distribution.
